# Dietary Exposure and Risk Assessment of Mycotoxins in Thyme and Thyme-Based Products Marketed in Lebanon

**DOI:** 10.3390/toxins14050331

**Published:** 2022-05-06

**Authors:** Hussein F. Hassan, Lara Koaik, André El Khoury, Ali Atoui, Tahra El Obeid, Layal Karam

**Affiliations:** 1 Department of Natural Sciences, School of Arts and Sciences, Lebanese American University, Beirut P.O. Box 13-5053, Lebanon; hussein.hassan@lau.edu.lb; 2 Department of Nursing & Health Sciences, Faculty of Nursing & Health Sciences, Notre Dame University-Louaize, Zouk Mikael P.O. Box 72, Lebanon; lmkoaik@ndu.edu.lb; 3 Centre d’Analyses et de Recherche (CAR), Unité de Recherche Technologies et Valorisation Agro-Alimentaire (UR-TVA), Faculty of Sciences, Saint-Joseph University of Beirut, Campus of Sciences and Technologies, Beirut P.O. Box 17-5208, Lebanon; andre.khoury@usj.edu.lb; 4 Laboratory of Microbiology, Department of Life and Earth Sciences, Faculty of Sciences, Lebanese University, Hadath Campus, Beirut P.O. Box 5, Lebanon; aatoui@ul.edu.lb; 5 Human Nutrition Department, College of Health Sciences, QU Health, Qatar University, Doha P.O. Box 2713, Qatar; tahra.e@qu.edu.qa

**Keywords:** thyme, AFB1, OTA, exposure, contamination

## Abstract

This study aimed at evaluating the incidence of aflatoxin B1 (AFB1) and ochratoxin A (OTA) in thyme and thyme-based products, related dietary exposure, and cancer risk for regular and high consumption. A total of 160 samples were collected, and 32 composite samples were analyzed. AFB1 and OTA were respectively found in 84% (27/32) and 38% (12/32) of the samples. AFB1 exceeded the limits in 41% (13/32) and 25% (8/32) of the samples according to the Lebanese and European standards, respectively. OTA was unacceptable in only 6% (2/32) and 3% (1/32) of the samples according to the Lebanese and European standards, respectively. AFB1 and OTA daily exposure was shown to be 4.270 and 1.345 ng/kg bw/day, respectively. AFB1 was shown to be associated with 0.41 and 0.35 additional cancer cases per 100,000 persons per year for regular consumption, respectively; while for high consumption, an increase of 0.911 and 0.639 cancer cases per 100,000 person per year was noted, respectively. The margin of exposure (MOE) for OTA was >10,000 for the non-neoplastic effect and >200 for the neoplastic effect, representing no toxicological concerns for consumers.

## 1. Introduction 

Mycotoxins are low-molecular-weight secondary metabolites of filamentous fungi mainly produced by *Alternaria*, *Aspergillus*, *Fusarium*, and *Penicillium* genera. They contaminate various agricultural commodities such as cereals and cereal products, nuts, dried fruits, spices, herbs, coffee, wine, and beer [1,2,3,4,5]. Mycotoxins can contaminate food and agricultural products at any stage of the food chain, including preharvest, harvest, drying, and storage stages, and their production increases mainly in areas of high temperature and humidity [6,7]. Moreover, they are chemically and thermally stable, making them more persistent in the food chain and during food preparation and transformation [8,9]. 

Aflatoxin B1 (AFB1) is the most common in foods, and is considered the most potent metabolite due to its genotoxic and carcinogenic characteristics [10]. Chronic exposure to AFB1 increases the risk of liver cancer, especially when associated with hepatitis B and C. The International Agency for Research on Cancer classified AFB1 as a Group 1 known human carcinogen [10]. Ochratoxin A (OTA), another major mycotoxin present in many foods, and also known for its nephrotoxicity, hepatotoxicity, teratogenicity, and immunotoxicity [11], was classified as a possible carcinogen to humans (Group 2B) [10]. 

Thyme is an edible wild plant native to the Mediterranean region and is considered to be one of the most important spice crops in world trade [12]. In the Eastern Mediterranean, what is collectively known thyme or “Zaatar” in Arabic refers to several species from the *Origanum*, *Thymus*, *Satureja*, and *Thymbra* genera [13,14]. Thyme is widely used in its fresh or dried forms for seasoning in baked goods, stews, meats, vegetables, salads, and marinades [13,14]. Moreover, thyme pizzas in different sizes and shapes (Mankooshe and Fatayer Zaatar) are very famous breakfasts or snacks [15]. In addition to its culinary uses, thyme has many health benefits. Thymol and carvacrol are two principal constituents of the thyme essential oil, and have been shown to have antioxidant, anti-inflammatory, respiratory, and neurological benefits [14]. Thyme is beneficial not only in culinary and medicinal uses, but also for the economy, since, for example, thyme mix and oregano exports from Lebanon reached 548 tons and a value of USD 1.9 million in 2014 [16].

However, thyme can be contaminated with AFB1 and OTA throughout the food chain, especially since the climate in the Mediterranean area is known for its high temperature and high humidity, especially in summer, which promote their production. Additionally, when stored improperly, thyme can be considered a good niche for fungal growth and mycotoxin contamination due to its high moisture content [17]. Previous studies reported mycotoxin occurrence in thyme [18,19,20]. Therefore, the Lebanese Standards Institution (LIBNOR) has set maximum limits (MLs) for thyme and thyme mixes of 2 μg/kg for AFB1 and 3 μg/kg for OTA [21]. The EU has no specific control measures for thyme. Instead, MLs are set for spices at 5 and 15 μg/kg for AFB1 and OTA, respectively [22]. The determination of those levels of contaminants is primary in assessing dietary exposure and the related adverse health effects on the population. Dietary exposure takes into consideration the concentration of the chemicals in the examined food, such as AFB1 and OTA, and the daily intake of the food item. Since ingestion of 1 ng/kg bw/day of AFB1 would induce 0.083 liver cancer cases per 100,000 persons per year, according to the Joint FAO/WHO Expert Committee on Food Additives [23], the ”as low as reasonably achievable” (ALARA) approach is recommended. For OTA, the European Food Safety Authority (EFSA) considered the previously used Provisional Tolerable Weekly Intake (PTWI) values no longer valid, and recommended the use of a margin of exposure (MOE) approach in the risk assessment of neoplastic and non-neoplastic effects [11]. As for the Joint FAO/WHO Expert Committee on Food Additives (JECFA), the PTWI of 100 ng/kg bw/week is still valid [24].

In order to evaluate the risk of exposure by consumers, it is important to assess not only the contamination of thyme, but also its related products, as it is a common ingredient in several recipes. Few studies have been conducted to evaluate AFB1 and OTA in food products in Lebanon [8,25,26,27,28]; however, no studies targeted thyme herbs and its related products specifically. The objective of the present study was to determine the occurrence of AFB1 and OTA, evaluate the dietary exposure, and assess the risk posed by AFB1 and OTA in thyme and thyme-based products marketed in Lebanon.

## 2. Materials and Methods

### 2.1. Food Selection and Sampling

Thyme herbs can be used in several homemade recipes and sprinkled over a variety of foods and dishes. For this purpose, a screening of the Lebanese market was carried out, and a list of commercialized thyme and related thyme-based products were selected to both be assessed in this study (Table 1). This list was used in a food-frequency questionnaire to estimate the consumption of thyme and thyme-based products by the Lebanese population, and the obtained data were used to estimate the dietary exposure to AFB_1_ and OTA, as well as the related risk assessment.

A total of 160 samples of thyme and thyme-based products were collected in two complete sets of different production dates at a six-month interval (September 2019–March 2020). A composite sampling approach was applied for each food sample. Five different brands of the same item were purchased and combined together with a 20% weight contribution for each brand to give a homogenized representative sample [8]. A total of 40 dried thyme herb samples were collected (4 dried thyme herb types × 5 different brands × 2 collections) and combined into 8 composite samples for analysis. A total of 120 thyme-based product samples were collected (12 thyme-based products types × 5 different brands × 2 collections) and combined into 24 composite samples for analysis. Thus, a total of 32 composite samples were prepared. Sampling was performed from representative and common retail markets in Lebanon. Cooked dishes (pasta, pizza, sandwiches) were prepared using traditional references, while the remaining food items were tested as ready to eat without any cooking or preparation. Samples were collected and stored at −18 °C prior to analysis. The equipment used for preparing and homogenizing the composite samples were thoroughly washed between each preparation to avoid the risk of cross-contamination. Although this composite method may have diluted the contamination concentration in each individual sample, it had the advantage of screening the overall market of thyme and thyme-based products by collecting a large number of samples [8].

### 2.2. Chemicals

The standard for AFB_1_ (2 µg/mL in acetonitrile) was purchased from Trilogy (Washington, USA), the standard for OTA (50 μg/mL in benzene:acetic acid 99:1) was purchased from Supelco (Bellefonte, PA, USA), the acetonitrile and methanol (HPLC grade) were purchased from Sigma-Aldrich (Steinheim, Germany), and the Aflaochra prep immunoaffinity columns specific to aflatoxins and OTA were purchased from RBiopharm Rhone Ltd. (Glasgow, Scotland, UK). Distilled water was used for the analysis, and all other chemicals and reagents were of analytical grade.

### 2.3. Sample Preparation 

According to R-Biopharm Rhone Ltd.’s extraction protocol, 25 g of the ground sample was blended with 5 g of sodium chloride and 100 mL of 80% methanol at a high speed for 2 min. Then, the samples were centrifuged at 4000 rpm for 10 min. Next, 2 ml of the filtrate was diluted with 18 mL of phosphate-buffered saline (PBS) and filtered through glass microfiber filter paper. After that, 10 mL of the filtrate was passed through the immunoaffinity columns at a slow steady flow rate to purify the toxins. Immunoaffinity columns were used previously to detect aflatoxins and ochratoxin A in spices [29,30]. The columns were then washed with 20 mL PBS to get rid of any sample residues. Finally, the toxins were eluted by passing 1 mL of methanol followed by 1 mL of distilled water to attain a final volume of 2 mL. The final eluted volumes were collected and stored in sealed vials at proper temperatures until the time of HPLC analysis.

### 2.4. HPLC Conditions

Reverse-phase high-performance liquid chromatography (HPLC) (Waters 2690^®^, Waters Corp., Burnsville, MN, USA) coupled with a fluorescence detector (Waters 2475^®^) and a Supelco Discovery^®^ HS C18 column (250 mm × 4.6 mm I.D., 5 μm particle diameter) fitted with a C18 guard column (Supelco Supelguard^®^, Sigma-Aldrich Co., St. Louis, MO, USA) at 25 °C was used for analysis. The method for the Aflaochra Prep immunoaffinity column from the supplier R-Biopharm Rhone Ltd. (specific for ochratoxin A and aflatoxin B1 purification from food matrices) was followed for HPLC analysis after optimization. Two mobile phases, which were prepared on the same day of the HPLC analysis, were used for analysis after filtering; solution A was composed of water:methanol (55:45 *v*/*v*), and solution B was composed of water:methanol (20:80 *v*/*v*). For each 1 L, 119 mg potassium bromide and 350 µL of 4 M nitric acid were added to both solutions. The mobile phases were passed at a flow rate of 0.8 mL/min. The sample injection volume was 100 μL, and the wavelengths for excitation and emission were 365 nm and 442 nm, respectively, from the start of the sample analysis until 17 min. Then, the excitation and emission were changed to 333 nm and 463 nm, respectively. A calibration curve was illustrated by using the aflatoxin B1 standard and ochratoxin A standard at concentrations ranging from 0 to 10 ppb. The performance of the method was tested using different parameters, including LOD (0.0042 and 0.0034 µg/kg for AFB1 and OTA, respectively), LOQ (0.027 and 0.015 µg/kg for AFB_1_ and OTA, respectively), and recovery analysis (88% and 94% for AFB_1_ and OTA, respectively). 

### 2.5. Exposure to AFB_1_ and OTA

In order to assess the exposure in the Lebanese population to AFB_1_ and OTA, a food-frequency questionnaire (FFQ) was developed to estimate the consumption of thyme and thyme products in Lebanon. A random representative sample of 1555 people was selected proportionally from 5 different Lebanese governorates (Beirut, Mount Lebanon, North, South, and Beqaa) to participate in this study. The FFQ assessed the regular and high consumption related to factors such as thyme cultivation season, lent meals, school snacks, picnic meals, weight-loss diet, and sickness remedies. After obtaining the consumption data, the exposure to AFB_1_ and OTA from thyme and thyme products was evaluated accordingly.

Daily dietary exposure to AFB_1_/OTA from thyme-based products or dried thyme herbs (ng/kg body weight/day) was calculated as: ((Daily intake (kg/day) × AFB_1_/OTA concentration (ng/kg))/ body weight. To estimate the daily dietary exposure, the average body weight of 72.4 kg obtained from the FFQ was used. The average daily exposure to AFB_1_ and OTA from each item was summed to obtain the total daily dietary exposure (ng/kg bw/day) from thyme-based products and dried thyme herbs in Lebanon.

According to recent studies highlighting the uncertainty of the mode of action of OTA for kidney carcinogenicity, comparing the dietary exposure to the health-based guidance values (HBGV) is no longer valid [11]. According to the aforementioned, the provisional tolerable weekly intake (PTWI) of 120 ng/kg bw/week previously set by EFSA also is no longer valid. The margin of exposure (MOE) approach was used for risk assessment for neoplastic effects and non-neoplastic effects of OTA. MOE was calculated as the ratio between the benchmark dose level (BMDL_10_) that causes a 10% increase in cancer incidence in animals and the daily dietary exposure. A BMDL_10_ of 4.73 µg/kg bw/day was used for non-neoplastic effects calculated from kidney lesions, and a MOE below 200 indicated a health risk. For neoplastic effects, a BMDL_10_ of 14.50 µg/kg bw/day was used that was calculated from kidney tumors in rats, and a MOE below 10,000 would indicate a public health concern [11]. However, according to the Joint FAO/WHO Expert Committee on Food Additives [24], the PTWI of 100 ng/kg bw/day was still considered.

For AFB_1,_ it was estimated that for non-European countries, the ingestion of 1 ng per kg of body weight per day of AFB_1_ would induce 0.083 liver cancer cases per 100,000 persons per year, according to the Joint FAO/WHO Expert Committee on Food Additives [23]. Thus, liver cancer risk based on total daily exposure to AFB_1_ (ng/kg body weight/day) from thyme and thyme-based products can be calculated as: liver cancer risk = daily exposure to AFB_1_ (ng/kg bw/day) × 0.083 cancer cases per 100,000 persons. The MOE approach also was used to assess the risk from AFB_1_ using a BMDL_10_ of 0.40 µg/kg bw/day for cancer incidence, based on rodent data as set by the CONTAM Panel. A MOE below 10,000 indicated a public health concern [31].

## 3. Results and Discussion

### 3.1. Occurrence of AFB_1_

The incidences of AFB_1_ and OTA in 32 samples of thyme and thyme-based products are shown in Table 2. AFB_1_ higher than the LOQ was found in 27 (87%) out of 32 composite samples, with a mean of 4.6 µg/kg and a concentration ranging between 0.08 and 25.8 µg/kg (Table 2). The maximum limit (ML) for thyme and thyme mixes according to the Lebanese Standards Institution (LIBNOR) is 2 µg/kg, compared to 5 µg/kg for spices as set by the European Commission [21,32]. The stricter limit by LIBNOR for thyme and thyme mixes is related to the different consumption habits and the higher amounts of thyme consumed and added to food formulations in the Mediterranean region, and in Lebanon especially. While dried thyme herbs can contribute to a high proportion of the product (1 to 35%) (Table 1), spices are generally added at much lower concentrations (10.4 g/portion) as flavoring agents to food [33]. Thyme is added to many dishes in Near Eastern cuisine, and contributes to many food products such as thyme pies, thyme salads, croissants, and many other breakfasts and snacks (Table 1) [15]. In addition, thyme is the most frequently eaten plant in Lebanon, at a frequency of five times per week [34]. However, according to the European regulations, thyme is considered to be in the spice category, and follows their limits (5 µg/kg), while knowing that the estimated consumption of spices and herbs in Europe (0.5 g/person/day) is lower than that in the Middle East (2.6 g/person/day) [35]. In addition, higher results were reported for the daily intake of dried thyme herbs in the current study, reaching 20.5 g/person/day (Table 1). For cereals, the ML set by LIBNOR is 2 µg/kg [36], the same as the European commission (2006) [32]. The AFB_1_ concentration exceeded the ML in 41% (13/32) and 25% (8/32) of the samples according to the Lebanese and European standards, respectively. Only 5 out of 24 thyme-based products were contaminated with AFB_1_ levels above the LIBNOR standards [21,36]. All dried thyme herb samples (8/8) exceeded the ML according to LIBNOR, compared to 6/8 samples exceeding the European regulations. For thyme-based products, thyme regular mix sandwich (2.55 µg/kg) and pizza and pasta (3.35 µg/kg) exceeded the ML according to both standards, while cheese with thyme was only unacceptable according to the Lebanese standards. The contamination range of cereal-based products in the current study (0.25–3.35 µg/kg) was higher than that found in the same category in Lebanon (0.005–0.827 µg/kg) [25], and in Turkey (0.013–0.178 µg/kg) [37]. The contamination range was also lower in a total diet study in Lebanon (0.010–0.260 µg/kg) [8]. In Saudi Arabia, all food items from bakeries were free from aflatoxin contamination [38]. In contrast to the current results, which showed the highest AFB_1_ concentration in pizza and pasta samples (3.35 µg/kg), all 27 pasta samples from Italy were free from AFB_1_ [39].

In the dried thyme herbs category, all the samples exceeded the limits, with thyme tea’s concentration reaching 6 times the Lebanese standards and 3 times the European standards. Thyme tea showed the highest AFB_1_ concentration of 16.79 µg/kg (Table 3). This high level was also shown in another study conducted on herbal green tea available in the Moroccan market, with a mean of 16.9 µg/kg in some areas [41]. In Pakistan and Latvia, AFB_1_ was also detected in tea samples in ranges of 0.08–8.24 µg/kg [42] and 3.40–23.7 µg/kg [43], respectively. In Italy, none of the herb tea samples were contaminated with AFB_1_ [44]. High levels of AFB1 in thyme dried herbs were also shown in other studies conducted in Lebanon. For instance, in one study, a mean of 193.4 µg/kg and 36.1 µg/kg was reported for spices and herbs [28], respectively. On the other hand, another study found a mean of 99.4 µg/kg for AFB1 in spices [27]. A range of 5.3–17 µg/kg of AFB_1_ contamination was shown in oregano in Serbia [45], and a mean of 16.8 µg/kg was reported in thyme in Egypt [46], which were comparable to our results. In Greece, 20 out of 29 spice samples were positive for AFB_1_, with a mean of 9.89 µg/kg and a range of 0.40–132.70 µg/kg [47]. Unlike our results, AFB_1_ was not detected in any of six thyme samples in a study conducted in Turkey [48], nor in a study in Italy [44]. In Iran, 1 out of 10 thyme samples was contaminated with AFB_1_, with a concentration of 2.2 µg/kg [49]. In Italy, the mean contamination of AFB_1_ in spices was 0.31 µg/kg, which was much lower compared to the current study [50]. These findings may be related to high counts of molds reported in thyme samples collected from the Lebanese market [51]. These molds included *Aspergillus*, which produces aflatoxins. Molds can contaminate spices and herbs due to bad agricultural practices at any stage during growth, harvesting, transportation, processing, or storage [17,52]. This highlights the importance of implementing food safety management systems, monitoring storage conditions, and applying good hygienic and manufacturing practices in the spices and herbs sector to ensure the microbiological quality of the products and prevent mold contamination [28,51]. 

AFB1 contamination in thyme-based products can be caused by the use of AFB1-contaminated ingredients, including dried thyme herb, wheat, oil, nuts, and seeds. While dried thyme herbs showed high AFB_1_ contamination levels (2.78–16.79 µg/kg), the contamination of the thyme-based products varied depending on the AFB_1_ concentration in the ingredients used in the final product (Table 3). Many other studies reported AFB_1_ contamination in several ingredients used in thyme-based products, such as wheat [25,53], thyme herb [27,28], nuts [54,55,56], and sesame [57,58].

### 3.2. Occurrence of OTA

OTA was found in 38% (12/32) of the samples, with a mean of 1.4 µg/kg and a concentration ranging between 0.04 and 8.00 µg/kg. Only 6% (2/32) and 3% (1/32) of the samples were contaminated with levels of OTA above the Lebanese and European standards, respectively (Table 2). All thyme-based products were below the limits of OTA for cereals and thyme, similar to a study in the US, which showed that breakfast cereals had generally low OTA levels, with a mean 1.51 µg/kg the first year and 0.60 µg/kg the second year [59]. In Spain, the OTA mean concentration in breakfast cereals was 0.265 µg/kg [60]. A study conducted in Lebanon to assess OTA in a 24 h diet in students showed a mean of 0.16 µg/kg. However, OTA was not detected in thyme manakish, nor in wheat products such as toast and tea [26]. Toast, croissant, and kaak samples in a study previously conducted in Lebanon [25] reported OTA concentrations of 4.11, 0.74, and 2.03 µg/kg, respectively. While croissant and kaak were free from OTA in our study, pasta and pizza samples in our study showed a concentration of OTA of 1.58 µg/kg, which was higher than that found in Turkey (0.065 µg/kg) and the Czech Republic (0.14 µg/kg) [61,62]. In Morocco, 125 pasta samples showed OTA mean concentrations of 0.63–0.64 ng/kg, which were much lower compared to the current study [63].

For the dried herbs category, only thyme regular mix exceeded the acceptable limit according to the Lebanese standards, with a concentration of 4.83 µg/kg. Other studies conducted in Lebanon reported higher OTA concentrations of 9.13 µg/kg [26] and 7.0–7.1 µg/kg [28] in spices and herbs, respectively. A study conducted in Italy also showed a higher OTA concentration in spices, with a mean of 6.18 µg/kg [50]. In Serbia, oregano was contaminated with OTA in a range of 4–22.4 µg/kg, which was much higher than in the current study [45]. In Poland, whole oregano and crushed oregano samples showed OTA means of 0.37 µg/kg and 1.77 µg/kg, respectively [64]. In Belgium, herbs and spices showed a mean of 1.51 µg/kg for OTA [65], lower than in the current study for dried thyme herbs. The OTA mean contamination in tea samples in this study was 0.07 µg/kg, with a range of 0.00–0.135 µg/kg; while in Latvia, a range of 2.99–30.3 µg/kg was reported [43]. In China, 2 out of 108 tea samples were positive for OTA, with an average of 0.66 µg/kg [66].

OTA incidence in food items in the current study was not only due to thyme, but also could be due to any of the raw materials in the food items, and could appear in the final products, since OTA is thermally stable [67]. For example, pizza and pasta showed an OTA concentration of 1.58 µg/kg, which may have been contributed by thyme, tomato sauce, wheat, or other raw materials that persisted after cooking. This highlighted the importance of setting limits for all raw materials used.

### 3.3. Risk Characterization and Dietary Exposure to AFB_1_

Table 4 shows the dietary exposure to AFB_1_ for regular and high consumption of thyme-based products and dried thyme herbs. For thyme-based products, the average daily exposures to AFB_1_ were 4.270 and 7.701 ng/kg bw/day for regular and high consumers, respectively. The dried thyme herbs category showed higher exposure values of 4.977 ng/kg bw/day and 10.980 ng/kg bw/day, respectively, for the same consumption levels.

The average dietary exposure values for AFB1 for regular consumption of thyme-based products and thyme dried herbs in the present study (4.270–4.977 ng/kg bw/day) were higher than those reported in a study on wheat products in Lebanon (0.92 ng/kg bw/day) [25]. In addition, our values were higher than what a Lebanese total diet study found for regular and excessive consumers (0.66 and 1.46 ng/kg bw/day, respectively) (8). In addition, our values were higher than those reported for spices used in Lebanese cooking (1.55 ng/kg bw/day) [27]. The exposure levels to AFB_1_ in this study were similarly higher than those reported in other countries. They were higher than the exposure from spices in Malaysia (0.59 ng/kg bw/day) [68], from breakfast cereals in Spain (0.01–0.06 ng/kg bw/day) [69], and from wheat flours in Iran (0.25 ng/kg bw/day) [70]. In China and Turkey, the dietary exposures to AFB_1_ from various food products were 0.57 ng/kg bw/day and 0.433 ng/kg bw/day, much lower compared to the current study [37,71]. Dietary exposure to AFB1 from a certain food depended on the dietary patterns and AFB1 levels in the food during the analysis period. The high concentration levels of AFB_1_ in thyme and thyme-based products, as well as the high consumption of these products by the Lebanese population, contributed to the high dietary exposure levels found in the current study. 

**Figure 1:**
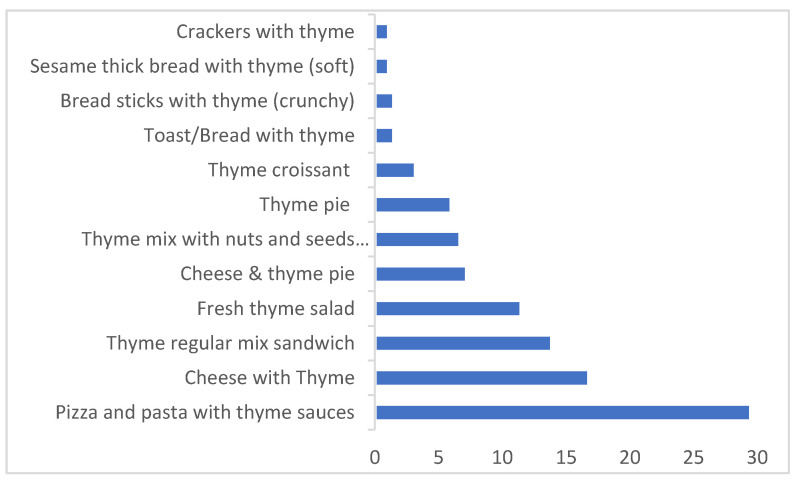
The percentage contributions of thyme-based products to the average dietary exposure to AFB_1_.

**Figure 2:**
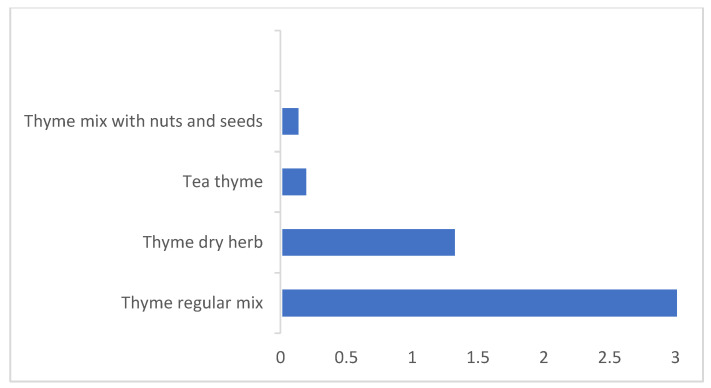
The percentage contributions of dried thyme herbs to the average dietary exposure to AFB_1_.

The main contributor to the dietary exposure to AFB_1_ in thyme-based products was pizza and pasta (30%); followed by cheese with thyme, thyme regular mix sandwich, and fresh thyme salad (17–12%); then cheese and thyme pie, thyme mix with nuts and seeds sandwich, and thyme pie (7–6%); with the remaining ones below 3% (Figure 1). For dried thyme herbs, thyme regular mix was the main contributor (66%), followed by thyme dry herb (27%), then thyme tea and thyme mix with nuts and seeds (3–4%) (Figure 2). For high consumption, the products’ contributions to the dietary exposure to AFB_1_ changed. The first three main contributors were cheese with thyme, pizza and pasta, and thyme regular mix sandwich (17–16%); followed by cheese and thyme pie, fresh thyme salad, and thyme mix with nuts and seeds (12–10%). This increase in the contributions of thyme in thyme regular mix sandwich, cheese and thyme pie, and thyme mix with nuts and seeds reflected the observed increase in thyme consumption during thyme cultivation season, lent meals, school snacks, and other meals in Lebanon.

In a study done in Lebanon, the main contributor to the average exposure to AFB_1_ was “bread and toast” (79.4%–82.2%) while “pizza and pies” contributed 4.9% to the dietary exposure to AFB_1_ [8]. The difference in the observed dietary exposure trend could be related to the differences among the products tested, variability in the contamination levels over time, and changes in consumers’ behavior due to several factors such as globalization, current economic crises, or other sociodemographic aspects. A study in New Zealand showed that spices were the major contributors to the dietary exposure to AFB_1_, followed by nuts, cereal products, and snacks [72].

The MOE to AFB_1_ determined using the dietary intake levels of each sample and the BMDL_10_ of 0.4 µg/kg bw/day [31] are shown in Table 5 and Table 6. 

The MOEs for thyme-based products for regular and high consumption were 94 and 52, respectively. These very low MOEs (less than 10,000) indicated a high risk due to consumption of thyme-based products, since the risk increased when the MOE decreased. On the individual product level, the lowest MOE was for pizza and pasta (317) followed by cheese with thyme (556), then thyme regular mix sandwich (675). For dried thyme herbs, the MOEs were 80 and 36 for regular and high consumption, respectively, highlighting the high daily intake and contamination of thyme herbs. As all the obtained MOE values were less than 10,000, the consumption of thyme and thyme-based products in Lebanon can put consumers at an exposure risk to AFB_1_.

No study regarding the MOE in thyme or thyme-based products was reported previously. However, the MOE calculated for other spices in past studies was similarly high. For example, the MOE to AFB_1_ through spices used in Lebanese cooking was reported to range between 108 and 444 [27]. In Malaysia, the MOE derived from mean exposure to AFB_1_ contamination in spices was reported to be 520 [68].

It is estimated that for non-European countries, the ingestion of 1 ng per kg of body weigh per day of AFB_1_ would induce 0.083 liver cancer cases per 100,000 persons per year, according to the Joint FAO/WHO Expert Committee on Food Additives [23]. Thus, liver cancer risk based on total daily exposure to AFB_1_ (ng/kg body weight/day) from thyme and thyme-based products can be calculated as: liver cancer risk= daily exposure to AFB_1_ (ng/kg bw/day) × 0.083 cancer cases per 100,000 persons. Therefore, according to the aforementioned, the Lebanese population would have 0.35 and 0.41 additional cancer cases per 100,000 persons per year for thyme-based products and dried thyme herbs, respectively. These corresponding values in the current study were higher than in studies conducted in Malaysia on spices (0.01) [68] and in Lebanon on wheat and products (0.076) [25]. For high consumption, this would mean an additional 0.911 and 0.639 cancer cases per 100,000 person per year for thyme and thyme-based products, respectively.

### 3.4. Risk Characterization and Dietary Exposure to OTA

The dietary exposure to OTA for regular consumption was calculated, and is shown in Table 5. The average total dietary intake of OTA was 1.345 ng/kg bw/day from thyme-based products, which was similar to another study conducted in Lebanon testing a 24 h diet showing a mean exposure of 1.4 ng/kg bw/day from the 24 h diet [26]. However, other studies in Lebanon reported higher exposures of 4.28 ng/kg bw/day [8] and 7.60 ng/kg bw/day [25]. In Turkey, a higher dietary exposure of 2.585 ng/kg bw/day from various food products was reported [61], while in the Czech Republic, it was comparable to the current study, with exposures of 1.8 and 1.2 ng/kg bw/day for adult men and women, respectively [62]. In addition, in Morocco, the average OTA daily exposure from cereal-derived products was 1.93 ng/kg bw/day, similar to our results [63]. The exposures to OTA from tea alone in the Czech Republic were 0.36 and 0.45 ng/kg bw/day for men and women, respectively, much higher than in the current study (0.001 ng/kg bw/day from thyme tea). The dietary exposure to OTA from dried thyme herbs was reported as 1.392 ng/kg bw/day compared to 0.0165 ng/kg bw/day for adults from herbs and spices in Belgium [65].

The estimated regular and high consumption weekly exposure levels to OTA from thyme-based products were 9.415 and 16.048 ng/kg bw/week, respectively (Table 5 and Table 6). Regular and high consumption exposure levels to OTA represented 9% and 16%, respectively, of the provisional tolerable weekly intake (PTWI) set by JECFA (100 ng/kg bw/week).

According to EFSA (2020), the PTWI of 120 ng/kg bw/week for OTA is no longer valid, after a recent study that reported uncertainty regarding the mode of action for kidney carcinogenicity [11]. Thus, the margin of exposure (MOE) approach was applied to assess the risk of OTA for both non-neoplastic and neoplastic effects. Both MOEs were calculated, and are shown in Table 6. The calculated MOE for regular consumption was above 10,000 for neoplastic effects and above 200 for non-neoplastic effects, which indicated in both cases that there was no potential health risk related to OTA from consumption of thyme and thyme-based products in Lebanon. The MOE in one study was reported to be below 10,000 for 24 h from meals and snacks for the neoplastic effect, indicating a major health threat; however, for the non-neoplastic effect, the MOE was above 200, which posed no threat [26]. In Belgium, the MOE showed a health concern due to the intake of biscuits, croissants, rice, flour, and herbs and spices [65]. On the other hand, the calculated MOEs for high consumption were above 200 for non-neoplastic effects. For neoplastic effects, all the MOE values were above 10,000 except for thyme regular mix (4818 < 10,000), indicating a risk from OTA intake for high consumption levels.

**Figure 3:**
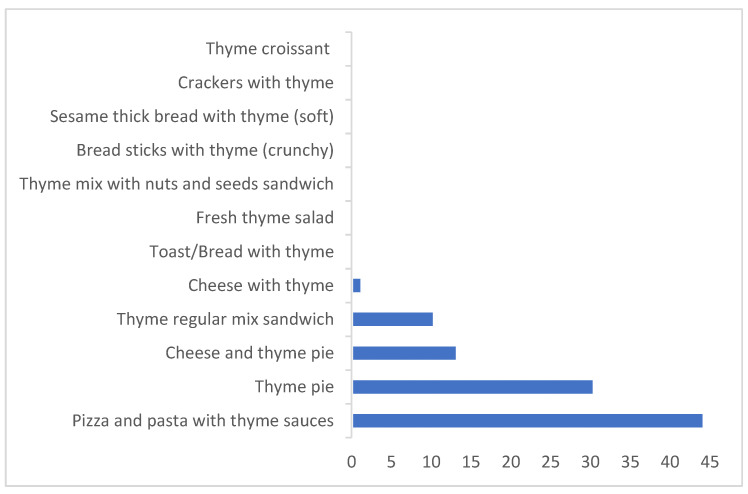
The percentage contributions of thyme-based products to the average dietary exposure to OTA.

**Figure 4:**
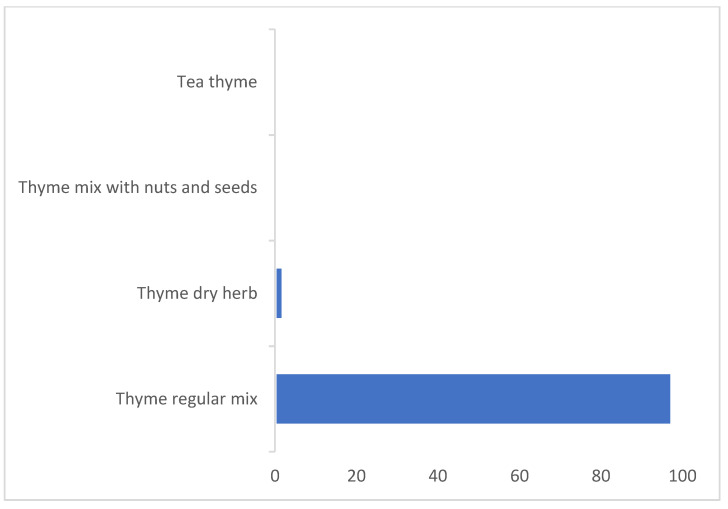
The percentage contributions of dried thyme herbs to the average dietary exposure to OTA.

The percentage contribution of each thyme-based product to the average dietary exposure to OTA was calculated (Figure 3). Pizza and pasta (44%) were the highest contributor to the dietary exposure to OTA, followed by thyme pie (32%), then cheese and thyme pie and thyme regular mix sandwich (13–11%), while the other products had a contribution below 1%. For high consumptions, thyme pie was the first contributor (35%), followed by pizza and pasta and cheese and thyme pie (27–24%), and then thyme regular mix sandwich (12%). Thyme regular mix was the major contributor to the exposure to OTA in the dried herbs category, with 98% (Figure 4). The main contributors to the dietary exposure to OTA in a total diet study previously conducted in Lebanon [8] were caffeinated beverages > biscuits and croissants > alcoholic beverages > bread and toast > rice. Cereal-based products were also the main contributors to OTA in European and North American areas [73].

## 4. Conclusions

In this study, the occurrence of AFB_1_ and OTA, dietary exposure, and risk assessment related to the consumption of thyme herbs and thyme-based products was investigated. Consumption of thyme and thyme-based products could lead to an increase in health risks associated mainly with AFB_1_, while no potential risk was associated with OTA. This is critical, especially since this food group is highly consumed in Lebanon and the Mediterranean area. This study highlighted the importance of developing maximum mycotoxin limits specific to each product and country. These limits must be based on risk assessment and consumption patterns. Our study also highlighted the importance of monitoring herbs to prevent exceeding the maximum limits to ensure the safety of the thyme and its products, especially since mycotoxins have the ability to resist processing. In addition, future studies should focus on testing individual samples, analyzing contamination trends over time, and assessing the effects of supply chain control and law enforcement on the contamination levels in thyme products.

## Figures and Tables

**Table 1:** Daily intake (g/day) of thyme-based products and dried thyme herbs for regular and high consumption obtained from a Food Frequency Questionnaire (FFQ) study in Lebanon.

		Daily Consumption (g/day)		Thyme Content % (*w*/*w*)
Food Items		Regular	High	
Thyme based products	Thyme pie	72.5	141.9	14
	Cheese and thyme pie	55.2	167.3	6
	Fresh thyme salad	68.2	115.8	4
	Thyme regular mix sandwich	16.8	34.0	31
	Thyme mix with nuts and seeds sandwich	11.2	29.2	35
	Pizza and pasta with thyme sauces	27.3	28.8	1
	Bread sticks with thyme (crunchy)	5.0	13.4	12
	Sesame thick bread with thyme (soft)	13.9	40.8	6
	Crackers with thyme	3.7	7.7	5
	Toast/bread with thyme	6.6	9.8	3
	Thyme croissant	9.6	23.0	9
	Cheese with thyme	20.9	38.8	1
Dried thyme herbs ^a^				
	Thyme regular mix ^a^	20.5	45.1	
	Thyme mix with nuts and seeds ^b^	3.9	7.1	
	Thyme herbs ^c^	3.9	10.1	
	Tea thyme	0.9	2	

The daily intake of: ^a^ thyme regular mix was estimated from thyme contribution to thyme pie, cheese and thyme pie, thyme regular mix sandwich, sesame thick bread with thyme (soft), and thyme croissant; ^b^ thyme mix with nuts and seeds was estimated from thyme contribution to thyme mix with nuts and seeds sandwich; ^c^ thyme herbs was estimated from fresh thyme salad, pizza and pasta with thyme sauces, bread sticks with thyme (crunchy), crackers with thyme, toast/bread with thyme and traditional molded aged cheese with thyme, and traditional strained yogurt balls with thyme.

**Table 2:** Incidence of AFB_1_ and OTA contamination in 32 thyme-based products and dried thyme herbs (µg/kg). ^a^.

Mycotoxin Type	Classification	Contamination Status N (%)
AFB_1_	No. of contaminated samples (%)	27 (84)
	0.08–2 µg/kg	14 (44)
	2–5 µg/kg	7 (22)
	>5 µg/kg	6 (19)
	Mean ± SD	4.6 ± 6.5
	Range	0.08–25.8
	No. of unacceptable samples (%)	
	Total	13/32 (41)
	Thyme-based products	5/24 (21) ^b^
	Dried thyme herbs	8/8 (100) ^b^/6 (75) ^c^
OTA	No. of contaminated samples (%)	12 (38)
	0.04–3 µg/kg	10 (31)
	3–15 µg/kg	2 (6)
	>15 µg/kg	0 (0)
	Mean ± SD	1.4 ± 2.3
	Range	0.04–8.0
	No. of unacceptable samples (%)	
	Total	2/32 (6)
	Thyme-based products	1/24 (4) ^d^
	Dried thyme herbs	1/8 (13) ^d^

^a^ Calculation of contamination mean and range was based on positive samples. All values are expressed in µg/kg. Unacceptable samples according to the following limits. ^b^ AFB1 maximum limits for Lebanese bread and thyme and thyme mixes (2 µg/kg) [21,36]. ^c^ AFB1 maximum limit for spices (5 µg/kg) [40]. ^d^ OTA maximum limits for Lebanese bread and thyme and thyme mixes (3 µg/kg) [21,36].

**Table 3:** Mean concentration (µg/kg) and ± SD of mycotoxins in thyme-based products (24 composite samples) and dried thyme herbs (8 composite samples) ^a^.

Food Items		AFB_1_	OTA
Thyme based products	Thyme pie	0.26 ± 0.36 ^b^	0.41 ± 0.58 ^d^
	Cheese and thyme pie	0.40 ± 0.57 ^b^	0.23 ± 0.33 ^d^
	Fresh thyme salad	0.52 ± 0.74 ^c^	0.00 ± 0.00 ^e^
	Thyme regular mix sandwich	**2.55 ± 2.08 ^b^**	0.60 ± 0.85 ^d^
	Thyme mix with nuts and seeds sandwich	1.85 ± 0.07 ^b^	0.00 ± 0.00 ^d^
	Pizza and pasta with thyme sauces	**3.35 ± 0.99 ^b^**	1.58 ± 2.03 ^d^
	Bread sticks with thyme (crunchy)	0.92 ± 1.30 ^b^	0.00 ± 0.00 ^d^
	Sesame thick bread with thyme (soft)	0.25 ± 0.35 ^b^	0.00 ± 0.00 ^d^
	Crackers with thyme	0.90 ± 0.79 ^b^	0.00 ± 0.00 ^d^
	Toast/Bread with thyme	0.69 ± 0.87 ^b^	0.02 ± 0.03 ^d^
	Thyme croissant	1.03 ± 0.33 ^b^	0.00 ± 0.00 ^d^
	Cheese with thyme	2.49 ± 0.50 ^c^	0.06 ± 0.09 ^e^
	Total	1.27 ± 0.75	0.24 ± 0.33
Dried thyme herbs			
	Thyme regular mix	**11.58 ^a^ ± 5.92 ^c^**	4.83 ± 4.44 ^e^
	Thyme mix with nuts and seeds	2.78 ± 0.06 ^c^	0.09 ± 0.13 ^e^
	Thyme dry herb	**15.38 ^a^ ± 0.30 ^c^**	0.35 ± 0.49 ^e^
	Thyme tea	**16.79 ^a^ ± 12.80 ^c^**	0.07 ± 0.10 ^e^
	Total	**11.63 ± 7.02**	1.34 ± 1.29

^a^ Mean was calculated as average of two collections. Mean was compared to: ^b^ AFB1 ML for Lebanese bread and for cereals and cereal products (2 µg/kg) [32,36]; ^c^ AFB1 ML for thyme and thyme mixes (2 µg/kg) [21] and for spices (5 µg/kg) [40]; ^d^ OTA ML for Lebanese bread, and for cereals and cereal products (3 µg/kg) [32,36]; ^e^ OTA ML for thyme and thyme mixes (3 µg/kg) [21], and for spices (15 µg/kg) [32]. Bold: exceeded the limits of the Lebanese standards and the European Commission. Underlined: exceeded the limits of the Lebanese standards only.

**Table 4:** Dietary exposure to AFB_1_ and the calculated MOE for regular and high consumption of thyme dried herb and thyme-based products marketed in Lebanon.

	Regular Consumption		High Consumption	
Thyme-Based Products	Exposure (ng/kg bw/day) ^a^	MOE ^b^	Exposure (ng/kg bw/day) ^a^	MOE ^b^
Thyme pie	0.257	**1554**	0.504	**794**
Cheese and thyme pie	0.306	**1308**	0.927	**432**
Fresh thyme salad	0.490	**817**	0.832	**481**
Thyme regular mix sandwich	0.592	**675**	1.198	**334**
Thyme mix with nuts and seeds sandwich	0.287	**1395**	0.748	**535**
Pizza and pasta with thyme sauces	1.262	**317**	1.332	**300**
Bread sticks with thyme (crunchy)	0.063	**6322**	0.170	**2359**
Sesame thick bread with thyme (soft)	0.047	**8474**	0.139	**2887**
Crackers with thyme	0.046	**8663**	0.096	**4163**
Toast/bread with thyme	0.063	**6352**	0.094	**4278**
Thyme croissant	0.136	**2932**	0.327	**1224**
Cheese with thyme	0.720	**556**	1.336	**299**
Total	4.270	**94**	7.701	**52**
Dried thyme herbs				
Thyme regular mix	3.280	**122**	7.216	**55**
Thyme mix with nuts and seeds	0.150	**2675**	0.272	**1469**
Thyme dry herb	1.339	**299**	2.146	**186**
Thyme tea	0.209	**1917**	0.464	**863**
Total	4.977	**80**	10.980	**36**

^a^ The average body weight of 72.4 kg was used. ^b^ BMDL10 of 0.4 µg/kg bw/day was used for MOE calculation of AFB1 [31]. Bold: MOE below 10,000 indicated a public health concern [31].

**Table 5:** Dietary exposure to OTA, margin of exposure (MOE), and the percentage contribution to toxicological reference values (TRVs) for thyme-based products and dried thyme herbs during regular consumption.

Thyme-Based Products	Exposure (ng/kg bw/day)	Exposure (ng/kg bw/week)	Dietary Exposure Expressed as %TRV JECFA ^a^	MOE Non-Neoplastic Effect ^b^	MOE Neoplastic Effect ^b^
Thyme pie	0.410	2.870	3	11,536	35,364
Cheese and thyme pie	0.179	1.250	1	26,478	81,170
Fresh thyme salad	0.000	0.000	0	-	-
Thyme regular mix sandwich	0.140	0.981	1	33,752	103,467
Thyme mix with nuts and seeds sandwich	0.000	0.000	0	-	-
Pizza and pasta with thyme sauces	0.596	4.171	4	7938	24,333
Bread sticks with thyme (crunchy)	0.000	0.000	0	-	-
Sesame thick bread with thyme (soft)	0.000	0.000	0	-	-
Crackers with thyme	0.000	0.000	0	-	-
Toast/bread with thyme	0.002	0.013	0	2,607,340	7,992,902
Thyme croissant	0.000	0.000	0	-	-
Cheese with thyme	0.018	0.127	0	260,685	799,140
Total	1.345	9.415	9	3517	10,781
Dried thyme herbs					
Thyme regular mix	1.368	9.575	10	3458	10,601
Thyme mix with nuts and seeds	0.005	0.035	0	958,357	2,937,880
Thyme dry herb	0.02	0.131	0	252,264	773,324
Thyme tea	0.001	0.006	0	5,637,070	17,280,658
Total	1.392	9.747	10	3398	10,417

^a^ OTA PTWI according to JECFA: 100 ng/kg/week [24]. ^b^ BMDL10 of 4.73 µg/kg bw/day for non-neoplastic effects and 14.50 µg/kg bw/day for neoplastic effects were used for MOE calculation of OTA [11]. MOE below 200 indicated a public health concern for non-neoplastic effects. MOE below 10,000 indicated a public health concern for neoplastic effects.

**Table 6:** Dietary exposure to OTA, margin of exposure (MOE), and the percentage contribution to toxicological reference values (TRVs) for thyme-based products and dried thyme herbs in high consumption.

Thyme-Based Products	Exposure (ng/kg bw/day) ^a^	Exposure (ng/kg bw/week)	Dietary Exposure Expressed as %TRV JECFA ^b^	MOE Non-Neoplastic Effect	MOE Neoplastic Effect
Thyme pie	0.803	5.618	6	5894	18,068
Cheese and thyme pie	0.541	3.790	4	8736	26,782
Fresh thyme salad	0.000	0.000	0	-	-
Thyme regular mix sandwich	0.284	1.985	2	16,677	51,125
Thyme mix with nuts and seeds sandwich	0.000	0.000	0	-	-
Pizza and pasta with thyme sauces	0.629	4.400	4	7524	23,066
Bread sticks with thyme (crunchy)	0.000	0.000	0	-	-
Sesame thick bread with thyme (soft)	0.000	0.000	0	-	-
Crackers with thyme	0.000	0.000	0	-	-
Toast/bread with thyme	0.003	0.019	0	1,755,964	5,382,975
Thyme croissant	0.000	0.000	0	-	-
Cheese with thyme	0.034	0.236	0	140,420	43,046
Total	2.293	16.048	16	2063	**6323**
Dried thyme herbs					
Thyme regular mix	3.009	21.065	21	1572	**4818**
Thyme mix with nuts and seeds	0.009	0.063	0	526,421	1,613,765
Thyme dry herb	0.049	0.340		97,409	298,610
Thyme tea	0.002	0.013	0	2,536,681	7,776,296
Total	3.069	21.481	21	1541	**4725**

^a^ OTA PTWI according to JECFA: 100 ng/kg/week [24]. ^b^ BMDL10 of 4.73 µg/kg bw/day for non-neoplastic effects and 14.50 µg/kg bw/day for neoplastic effects were used for MOE calculation of OTA [11]. Bold: MOE below 200 indicated a public health concern for non-neoplastic effects. MOE below 10,000 indicated a public health concern for neoplastic effects.

## Data Availability

Not applicable.
